# Controllable Synthesis of Mn_3_O_4_ Nanowires and Application in the Treatment of Phenol at Room Temperature

**DOI:** 10.3390/nano10030461

**Published:** 2020-03-04

**Authors:** Runlin Han, Min Chen, Xiaobing Liu, Yuhang Zhang, Yongli Xie, Yan Sui

**Affiliations:** 1 School of Chemistry and Chemical Engineering, Jinggangshan University, Ji’an 343009, China; c.m03@mail.scut.edu.cn (M.C.); liuxiaobing805@163.com (X.L.); 2 School of Chemical Engineering, Dalian University of Technology, Dalian 116024, China; zhangyuhang666@mail.dlut.edu.cn (Y.Z.); lililu@mail.dlut.edu.cn (Y.X.)

**Keywords:** manganese oxide, phenol treatment, nanowire, hydrothermal method, oxidation

## Abstract

Nanosized Mn_3_O_4_ nanowires are prepared with KMnO_4_ and ethanol in mild conditions by facile hydrothermal method. Hydrothermal reaction temperature is optimized to get uniform nanowires. The prepared Mn_3_O_4_ nanowires exhibit high activity in the treatment of phenol at acid condition and room temperature. The 20 mg Mn_3_O_4_ nanowires can efficiently dispose of 50 mL phenol solution (0.2 g·L^−1^) at pH 2 and 25 °C. The nanowires before and after phenol treatment are characterized by scanning electron microscopy (SEM), X-ray diffraction (XRD) and X-ray photoelectron spectroscopy (XPS) and the reaction mechanism is discussed.

## 1. Introduction

Manganese is one of the most earth-abundant elements and manganese oxides are generally non-toxic. Manganese oxide nanomaterials also show great potential in sustainable nanotechnology. They are widely utilized in catalytic reactions, sensors and batteries because of their low cost and high activity [1,2]. Mn_3_O_4_, also known as hausmannite, is a mixed valence oxide and a promising candidate for catalysts, microwave absorption materials, sensors and anode materials [3]. It has been used in the catalytic oxidation of methane and reduction of nitrobenzene [4]. Lu et al. prepared the amorphous MnO_2_ thin film first then hydrothermally transformed it into Mn_3_O_4_ nanowires under room temperature in a solution bath of 0.01 M manganese acetate and 0.01 M sodium sulfate mixture [5]. One-dimensional (1D) nanostructures, especially nanowires, are of great importance because of their specific shape and potential applications. Nanowires feature superior functional properties and mechanical strengths which have been used in micro/nanoelectromechanical systems and photovoltaic applications [6]. Veeramani et al. presented a room-temperature synthesis of Mn_3_O_4_ nanowires solely from water and manganese salt, catalyzed by iron oxide nanocrystals in the presence of piperazine-N,N^I^-bis(2-ethanesulfonic acid) (PIPES) [7]. Sambasivam et al. synthesized single-crystalline Mn_3_O_4_ nanowires using solvothermal technique [8].

Tremendous discharge of toxic organic compounds has contributed to serious pollution to our eco-environment and human beings. Phenolic compounds play an important role in the production of pesticides, resins and antioxidants [9]. However, the phenolic waste waters are highly toxic and persistent, and are difficult to treat with traditional biodegradation. Strong oxidants such as H_2_O_2_ and O_3_, without secondary pollution, have been utilized in the treatment of organic compound waste waters. However, the treatment efficiency is relatively low [10,11]. To achieve high mineralization, advanced oxidation processes (AOPs) were proposed to degrade organic pollutants through the generation of highly reactive hydroxyl radicals [12,13]. The Fenton process combines H_2_O_2_ and iron as a catalyst to generate hydroxyl radicals, which have strong oxidation capability on the phenol removal [14]. However, the homogeneous Fenton process is limited by its narrow pH range and iron sludge generation [15,16,17].

In this work, uniform Mn_3_O_4_ nanowires are fabricated with facile hydrothermal method and common raw materials in mild conditions. They are utilized to deal with phenol solution without extra H_2_O_2_ or O_3_ at room temperature and air condition. The mechanism of the Mn_3_O_4_ nanowires on the treatment of phenol is also studied with the characterization of scanning electron microscopy (SEM), X-ray diffraction (XRD) and X-ray photoelectron spectroscopy (XPS).

## 2. Experimental

### 2.1. Preparation and Characterization of Mn_3_O_4_

The nanosized Mn_3_O_4_ was provided by US research Nanomaterials, Inc (Houston, TX, USA) for comparison. Analytically pure KMnO_4_ and ethanol was used as raw materials. First, 0.2 g KMnO_4_ and 20 mL ethanol/water solution were added in a 45 mL Teflon-lined steel autoclave. The ratio of ethanol and reaction temperature in the furnace are investigated to get uniform nanowire and improved performance in phenol treatment. All products were cleaned with deionized water several times and dried in air at 80 °C for 12 h. Then the Mn_3_O_4_ powder was ground before utilization. The 3Flex Surface Characterization Analyzer (Micromeritics Corporation, Norcross, GA, USA) is able to determine the adsorption–desorption isotherm of nitrogen at 77 K, then, as a function of the kind of isotherm, according to international union of pure and applied chemistry (IUPAC) classification, a mathematical model to find specific surface areas (e.g., Brunauer–Emmett–Teller (BET) adsorption). The prepared Mn_3_O_4_ and used nanoparticles were tested with SEM (FE-SEM, Hitachi S-4800, Tokyo, Japan), XPS (ThermoESCALAB250Xi, Waltham, MA, USA) and XRD (XRD-7000S, Shimadzu, Tokyo, Japan) to determine the morphology and chemical composition of the material.

### 2.2. Treatment of Phenol Waste Water at Room Temperature

The phenol was dissolved in de-ionized water with H_2_SO_4_ to adjust the pH. In a typical procedure, 20 mg Mn_3_O_4_ was added into a phenol solution (50 mL, 0.2 g·L^−1^ and pH = 2) at room temperature in an air flow. The concentration of phenol is tested with UV-Vis spectrum at 270 nm every 15 min after the reaction solution was filtrated with a 0.1 μm poly tetra fluoroethylene (PTFE) syringe filter (Whatman^TM^, Shanghai, China).

## 3. Results and Discussion

### 3.1. Composition and Morphologies of the Prepared Mn_3_O_4_

**Figure 1:**
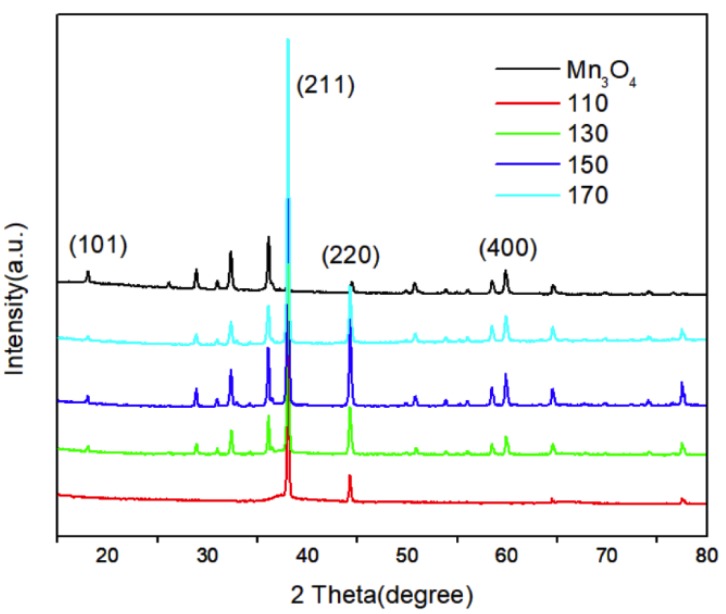
X-ray diffraction patterns of prepared particles at various temperatures for 4 h. (From top to bottom: Commercial nanosized Mn_3_O_4_; 170; 150; 130; 110 °C).

The effect of hydrothermal temperature on the diffraction patterns are shown in Figure 1. It is obvious that the prepared nanoparticles are all Mn_3_O_4_ which are similar with the commercial Mn_3_O_4_ nanoparticles. What is more, they are all in accordance with hausmannite type Mn_3_O_4_ PDF card (JCPDS no. 24-0734) even though the intensity of some peaks are different which may be induced by the formation of nanowires. When the reaction temperature decreases to 110 °C, the intensity of (211) plane is very high while some other planes such as (101), (220) and (400) planes almost disappeared.

**Figure 2:**
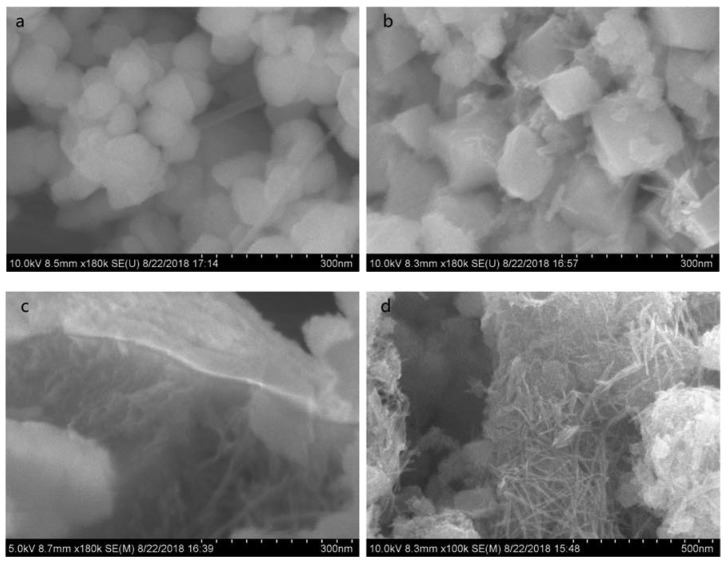

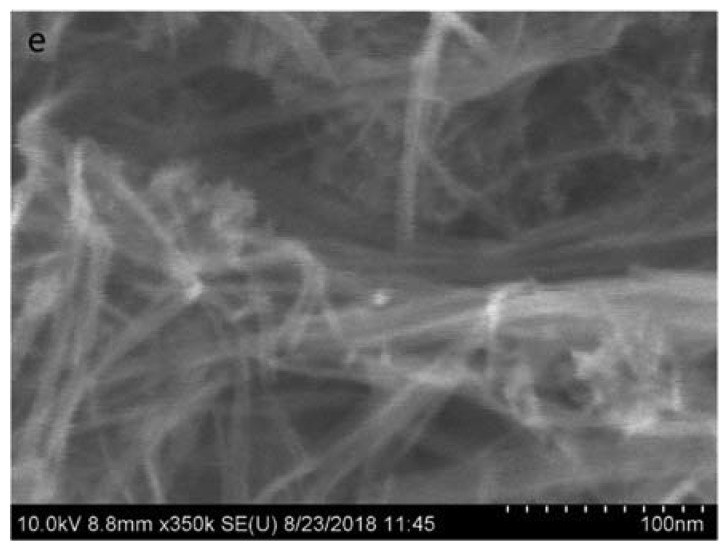
Scanning electron microscopy (SEM) images of Mn_3_O_4_ synthesized at different temperatures for 4h (**a**) commercial Mn_3_O_4_; (**b**) 170; (**c**) 150; (**d**) 130; (**e**) 110 °C.

The SEM images of Mn_3_O_4_ prepared at different temperatures are shown in Figure 2. When the hydrothermal temperature is 170 °C, uniform octahedron-like Mn_3_O_4_ particles with edge size about 60 nm are observed which are similar with the commercial Mn_3_O_4_ nanoparticles. With the decrease of temperature, the uniform Mn_3_O_4_ nanowires appear while the blocks disappear gradually. The nanowires with large specific surface area will improve the performance of Mn_3_O_4_. According to the data of BET surface area test, the BET surface area of commercial Mn_3_O_4_ is 14.48 m^2^·g^−1^ while the BET surface area of Mn_3_O_4_ (110 °C) and Mn_3_O_4_ (130 °C) are 248.8 and 189.69 m^2^·g^−1^, respectively, as shown in Table 1. Along with this, the adsorption average pore diameter of Mn_3_O_4_ (110 °C) is about 10.6 nm which is positive to the adsorption and reaction activity of the nanowires.

### 3.2. Treatment of Phenol with Mn_3_O_4_ at Room Temperature

**Figure 3:**
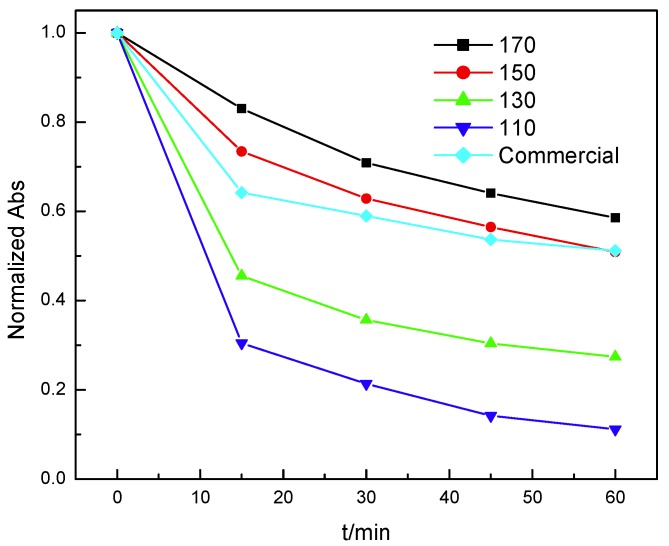
Effect of preparation temperature on the performance of Mn_3_O_4_ (From top to bottom: 170; 150; 130; 110 °C and commercial Mn_3_O_4_).

Phenol is widely used in the manufacturing of plastics, pesticides and pharmaceuticals. Because of its high toxicity, a lot of method is recommended but still not efficient. In this work, nanosized Mn_3_O_4_ (20 mg) was used to deal with the phenol waste water at room temperature in the air condition. As shown in Figure 3, the phenol concentration decreases obviously in 60 min. Especially when the preparation temperature is 110 °C, the performance of the Mn_3_O_4_ is the best because the Mn_3_O_4_ prepared at 110 °C shows the uniform nanowires and highest specific surface area. As shown in Table 2, the Mn_3_O_4_ nanowires can efficiently deal with phenol waste water with high concentration at low temperature.

**Figure 4:**
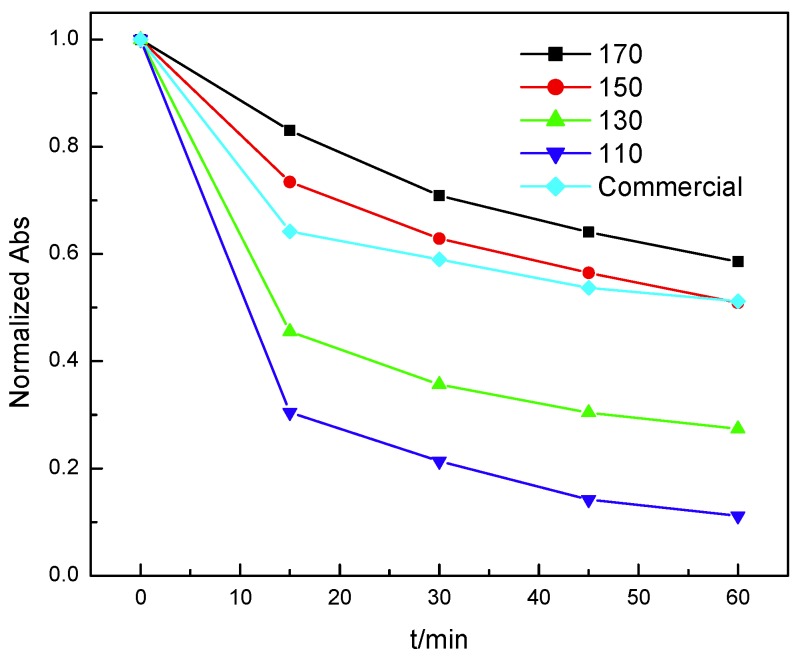
Effect of pH on the performance of Mn_3_O_4_.

Effect of pH on the performance of Mn_3_O_4_ is studied as shown in Figure 4. At neutral condition, almost no phenol will be consumed at all, which means that Mn_3_O_4_ will not react with phenol or adsorb it. With the decrease of pH, the reactivity of Mn_3_O_4_ increases obviously. When the pH is below 2, the Mn_3_O_4_ has good performance on treatment of phenol. However, lower pH will cause serious leaching of Mn_3_O_4_ because the nanosized Mn_3_O_4_ can be dissolved in concentrated sulfuric acid and hydrochloric acid.

### 3.3. Analyse of the Function of Mn_3_O_4_ in the Treatment of Phenol

**Figure 5:**
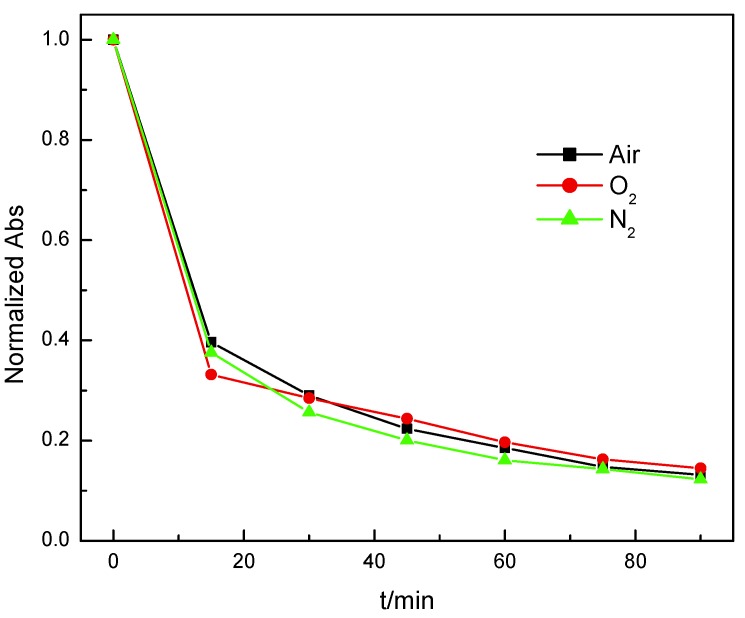
Effect of reaction atmosphere on the performance of Mn_3_O_4_.

Different reaction atmosphere is utilized to investigate the mechanism of phenol treatment. As shown in Figure 5, in all the reaction condition, the Mn_3_O_4_ has the similar performance which means oxygen is not necessary for the treatment of phenol waste water. In the product of the reaction, benzoquinone was detected which means Mn_3_O_4_ nanowires can oxidize phenol at room temperature.

H_2_O_2_ is an effective oxygen content with low cost and environmentally friendly nature. The excess H_2_O_2_ can decompose into safe H_2_O and O_2_ [20]. However, H_2_O_2_ (0.1 mol·L^−1^) cannot oxidize phenol at the same pH in air condition. In contrast, Mn_3_O_4_ nanowires prepared in this work with large specific area has stronger oxidation reactivity compared with H_2_O_2_ because it can oxidize H_2_O_2_. After reaction with H_2_O_2_, oxygen is formed while Mn_3_O_4_ is dissolved in the solution. KMnO_4_ which is the raw material of Mn_3_O_4_ nanowires also have strong oxidation ability. In order to completely oxidize phenol, excess dosage of KMnO_4_ is needed which will induce secondary pollution to the waste water. Excess Mn_3_O_4_ nanowires can be easily filtrated and leaching Mn ions can also be removed by neutralization of the acidic waste water.

**Figure 6:**
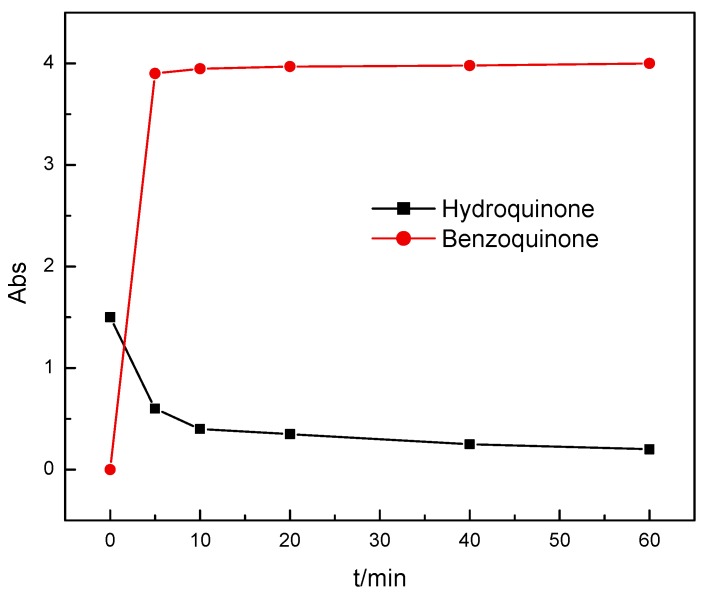
Effect of reaction time on the concentration of hydroquinone and benzoquinone.

Benzoquinone and hydroquinone are the common oxidation products of phenol. In this work, pure benzoquinone and hydroquinone solutions are used as the waste water separately, while 20 mg Mn_3_O_4_ is added in the 50 mL solution (pH = 2, adjusted with H_2_SO_4_). After 5 min, about 60% hydroquinone is converted to benzoquinone as shown in Figure 6. It was also found that the solution is changed into yellow (the color of benzoquinone) while all Mn_3_O_4_ nanowires are dissolved which demonstrates that hydroquinone can react with the nanoparticles efficiently. For the benzoquinone solution, the absorbance does not change which means that the Mn_3_O_4_ nanowires cannot react with stable benzoquinone.

**Figure 7:**
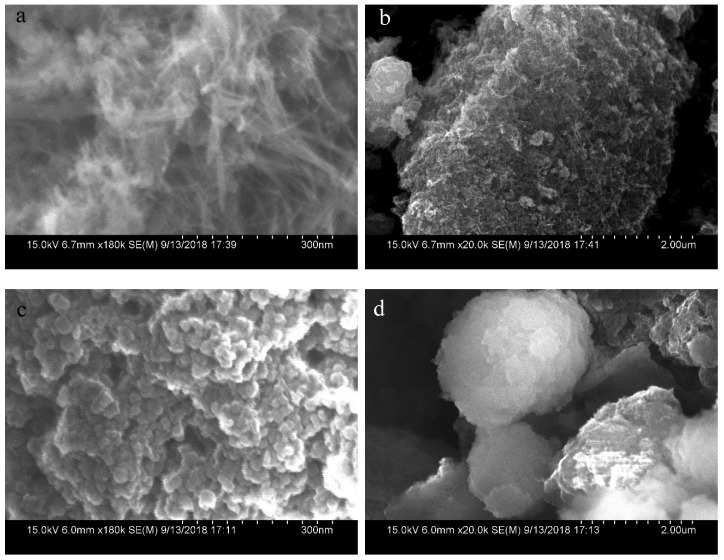
The morphology changes of Mn_3_O_4_ before and after reaction with phenol (**a**) Mn_3_O_4_ before reaction with magnification of 180,000×; (**b**) Mn_3_O_4_ before reaction with magnification of 20,000×; (**c**) Mn_3_O_4_ after reaction with magnification of 180,000×; (**d**) Mn_3_O_4_ after reaction with magnification of 20,000×.

After reaction with phenol, the morphology of Mn_3_O_4_ changes obviously as shown in Figure 7. The uniform nanowires disappear while a lot of block-shaped particles are formed. After reaction, the reactivity of Mn_3_O_4_ also disappears because the reaction will not continue after adding extra phenol.

**Figure 8:**
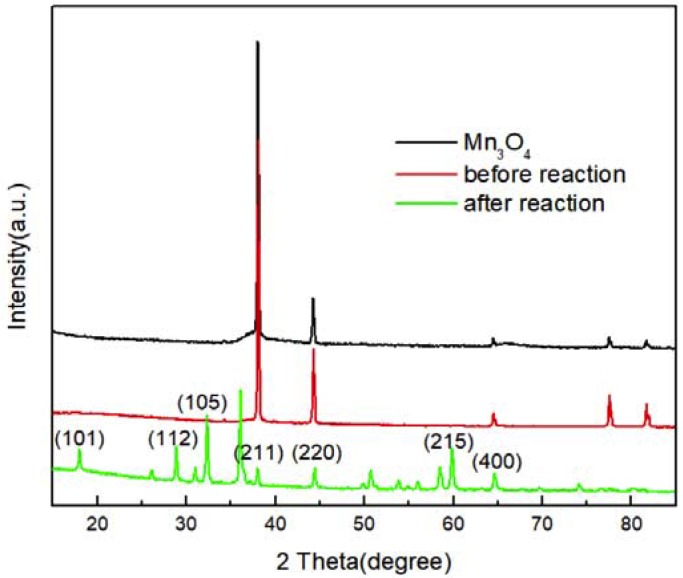
XRD patterns of Mn_3_O_4_ before and after reaction with phenol.

Although the morphology of the Mn_3_O_4_ particles changes obviously after reaction with phenol, the XRD patterns of Mn_3_O_4_ after reaction as shown in Figure 8 does not change very much and the characteristic peaks keep similar intensity which means only partial active surface area reacts with phenol. The reacted Mn_3_O_4_ still has the similar crystal structure with hausmannite.

**Figure 9:**
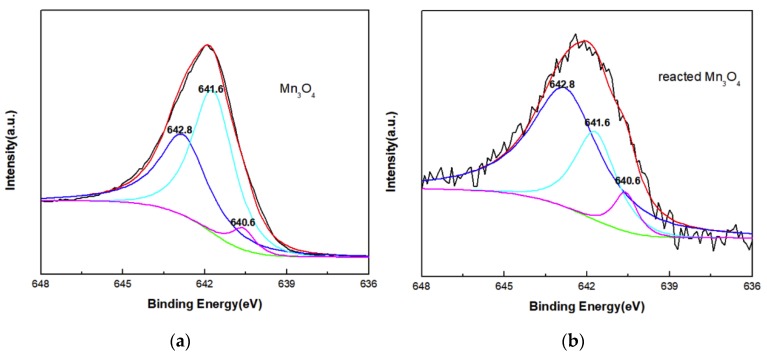
Mn 2p spectra XPS of Mn_3_O_4_ before and after reaction with phenol (**a**) prepared Mn_3_O_4_; (**b**) reacted Mn_3_O_4_.

The chemical composition and element valence of the materials before and after phenol removal are further examined by XPS (Figure 9). The Mn 2p3/2 peak of the prepared Mn_3_O_4_ and reacted Mn_3_O_4_ consisted of three separate peaks at 642.8, 641.6 and 640.6 eV (Figure 9b) which are in accordance with Mn^4+^, Mn^3+^ and Mn^2+^, respectively. Before reaction, the relative atomic percentages of Mn^4+^, Mn^3+^ and Mn^2+^ are 41.9%, 53.5% and 4.6%, respectively. After reaction with phenol, the relative percentages change into 68.1%, 25.5% and 6.4%. Although the average valence is improved according to the data, the possible reason is the dissolution of Mn with low valence in acid condition because the XPS spectrum of reacted Mn_3_O_4_ shows obvious noise which indicates pollution of the sample and low content of Mn.

## 4. Conclusions

Hydrothermal temperature and ethanol content have a strong effect on the morphology and structure of nanoparticles. Uniform Mn_3_O_4_ nanowires with high specific surface area (BET surface area, 248.8 m^2^·g^−1^) are formed when the hydrothermal temperature is 110 °C and the ethanol content is 10 mL. The Mn_3_O_4_ nanowires show high efficiency in phenol removal at room temperature and air condition because it has strong oxidation ability compared with H_2_O_2_. The 20 mg Mn_3_O_4_ nanowires can efficiently dispose of 50 mL phenol solution (0.2 g·L^−1^) at pH 2 and 25 °C. According to the SEM, XRD and XPS characterization, the Mn_3_O_4_ shows strong oxidation capability and reacts with phenol. After reaction, partial Mn with low valence is leaching into the acid solution. Therefore, it is very efficient in the quick removal of phenol in water treatment without special operation parameter. Excess Mn_3_O_4_ nanowires can be easily filtrated and leaching Mn ions also can be removed by neutralization of the acidic waste water.

## Figures and Tables

**Table 1:** BET surface area of Mn_3_O_4_.

	Mn_3_O_4_ (Commercial)	Mn_3_O_4_ (130 °C)	Mn_3_O_4_ (110 °C)
BET surface area/(m^2^·g^−1^) Adsorption average pore diameter/nm	14.48 21.5	189.69 10.3	248.8 10.6

**Table 2:** Comparison of phenol removal with different reagents.

Reagent and Content	Phenol Concentration	Temperature /°C	Time /h	Conversion /%	References
2.5 g·L^−1^ Au/C + 5 mL H_2_O_2_	45 mL (5 g·L^−1^)	80	22	~100	[18]
20 mg CNT/PEG	50 mL(20 mg·L^−1^)	120	0.5	~98	[19]
20 mg Mn_3_O_4_ nanowires	50 mL (200 g·L^−1^)	25	1	93	This work
